# Regional myocardial function after intracoronary bone marrow cell injection in reperfused anterior wall infarction - a cardiovascular magnetic resonance tagging study

**DOI:** 10.1186/1532-429X-13-22

**Published:** 2011-03-17

**Authors:** Einar Hopp, Ketil Lunde, Svein Solheim, Svend Aakhus, Harald Arnesen, Kolbjørn Forfang, Thor Edvardsen, Hans-Jørgen Smith

**Affiliations:** 1 Department of Radiology, Oslo University Hospital, Rikshospitalet, Postbox 4950, Nydalen, 0424 Oslo, Norway; 2 Department of Cardiology, Oslo University Hospital, Rikshospitalet, Norway; 3 Department of Cardiology, Oslo University Hospital, Ullevål, Norway; 4 Faculty of Medicine, University of Oslo, Oslo, Norway

## Abstract

**Background:**

Trials have brought diverse results of bone marrow stem cell treatment in necrotic myocardium. This substudy from the Autologous Stem Cell Transplantation in Acute Myocardial Infarction trial (ASTAMI) explored global and regional myocardial function after intracoronary injection of autologous mononuclear bone marrow cells (mBMC) in acute anterior wall myocardial infarction treated with percutaneous coronary intervention.

**Methods:**

Cardiovascular magnetic resonance (CMR) tagging was performed 2-3 weeks and 6 months after revascularization in 15 patients treated with intracoronary stem cell injection (mBMC group) and in 13 controls without sham injection. Global and regional left ventricular (LV) strain and LV twist were correlated to cine CMR and late gadolinium enhancement (LGE).

**Results:**

In the control group myocardial function as measured by strain improved for the global LV (6 months: -13.1 ± 2.4 versus 2-3 weeks: -11.9 ± 3.4%, p = 0.014) and for the infarct zone (-11.8 ± 3.0 versus -9.3 ± 4.1%, p = 0.001), and significantly more than in the mBMC group (inter-group p = 0.027 for global strain, respectively p = 0.009 for infarct zone strain). LV infarct mass decreased (35.7 ± 20.4 versus 45.7 ± 29.5 g, p = 0.024), also significantly more pronounced than the mBMC group (inter-group p = 0.034). LV twist was initially low and remained unchanged irrespective of therapy.

**Conclusions:**

LGE and strain findings quite similarly demonstrate subtle differences between the mBMC and control groups. Intracoronary injection of autologous mBMC did not strengthen regional or global myocardial function in this substudy.

**Trial registration:**

ClinicalTrials.gov: NCT00199823

## Background

Different studies have brought diverse results on the effects of cell therapy in acute myocardial infarction. Effect measures have included clinical parameters and measurements of global left ventricular (LV) function obtained through a spectrum of methods[1-6]. Some groups have explored regional left ventricular function as evaluated by wall motion or wall thickening assessed from cardiovascular magnetic resonance (CMR) or myocardial remodeling assessed from strain echocardiography[4,7-10]. Treatment for large myocardial infarctions is considered more challenging due to risk for LV dilatation and progressive ejection fraction (EF) reduction, and in three of the studies subgroup analyses have indicated a more substantial beneficial effect from stem cell therapy in large infarctions or in hearts with low LV EF[8,9,11]. Myocardial strain calculated from CMR tagging is currently regarded as the non-invasive gold standard for assessment of regional function. However, limited availability and analysis effort seem to reduce the overall use of the method[12,13]. In this substudy of the Autologous Stem Cell Transplantation in Acute Myocardial Infarction trial (ASTAMI)[2], we calculated LV circumferential strain and twist from short axis grid CMR tagging obtained on 28 patients first at 2-3 weeks and then subsequently 6 months after the infarction. We examined whether intracoronary injection of autologous mononuclear bone marrow cells (mBMC) influenced regional myocardial function or LV twist. In addition, we aimed to explore the potentials for tagging analysis to detect more subtle changes in myocardial function undetectable by other examination techniques in routine use.

## Methods

### Study group

Methods and techniques used in the ASTAMI trial have been reported in detail previously[2]. Briefly, 100 patients with acute left anterior descending artery (LAD) myocardial infarction were randomized to either intracoronary injection of autologous mBMC (mBMC group) or control with no sham injection after successful revascularization. mBMC injection was performed 4-8 days (mean 6 days) after percutaneous coronary intervention (PCI). Baseline CMR was performed after 2-3 weeks (18.8 ± 3.8 days) after myocardial infarction and was repeated after 6 months. For this substudy, the CMR protocol included short axis grid tagging sequences in addition to cine images and post contrast late gadolinium enhancement (LGE). The patients were a consecutive series of the last 28 patients included in ASTAMI who either received mBMC per protocol (n = 15) or belonged to the control group (n = 13). The study complies with the Declaration of Helsinki, and the protocol was approved by the regional committee for research ethics. All patients gave written, informed consent.

### Cardiac Magnetic Resonance

Cine images, tagging sequences and late enhancement images were acquired in the same image session with 1.5 tesla units (Magnetom Vision Plus or Magnetom Sonata, Siemens, Erlangen, Germany) with a phased array body coil.

Two cine long axis projections of the left ventricle were acquired with either a breath-hold segmented spoiled gradient echo sequence, fast low angle shot (FLASH) or a breath-hold segmented balanced gradient echo sequence, fast imaging with steady-state free precession (trueFISP). Each patient had either paired FLASH or paired trueFISP examinations. Temporal resolution was 50 ms or less and slice thickness was 6 mm.

Tagged CMR of the left ventricle was obtained with a FLASH sequence. Three short axis levels were standardized with the basal level just apical to the mitral ring at end-systole, the mid-ventricular level on the mid-point of the left ventricular long axis, and apical level just basal to the level of luminal closure at end-systole. Orthogonal tags in a grid pattern were parallel or perpendicular to the 2-chamber long axis plane with distance between tags of 8 mm. Temporal resolution was less than 50 ms and slice thickness 6 or 8 mm.

Late enhancement images were obtained 10 - 20 minutes after intravenous injection of 0.2 mmol/kg gadopentetate dimeglumine (Magnevist, Schering, Berlin, Germany) in two long axis projections corresponding to the cine images and multiple short axis projections covering the LV with a breath-hold inversion recovery turbo gradient echo sequence. The inversion time was chosen to null the signal of the normal myocardium. Slice thickness was 7 or 8 mm and increment between slices was 10 mm.

### CMR analysis

All CMR analyses were performed blinded to treatment allocation. End diastolic volumes (EDV), end systolic volumes (ESV) and EF were calculated according to the biplane area-length method from the two long axis cine projections[14].

**Figure 1:**
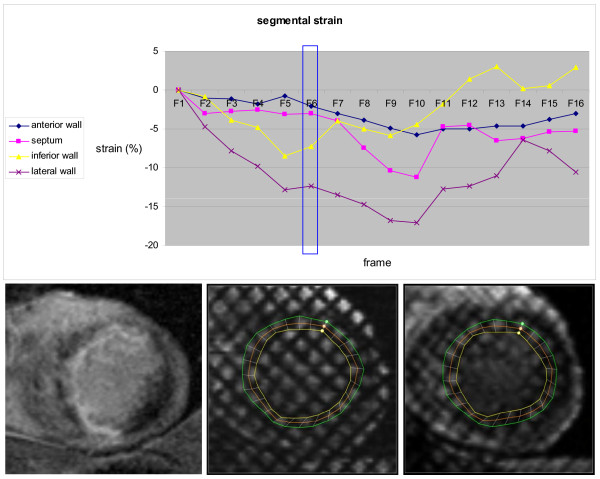
**Baseline apical tagging analysis in a 49 year old male patient in the mBMC group**. Upper part: The curves represent strain in the apical segments, analyzed with HARP software from a short axis CMR tagging sequence. The vertical box represents the end systolic frame (F6). There is almost normal function in the lateral wall, dysfunction in the inferior wall, while septum and the anterior wall are almost akinetic with possible post-systolic contraction. Note increasing noise during diastolic frames, due to T1 relaxation of the tagged myocardium. Lower part: Left: Short axis apical LGE image with hyperintensity in the anterior wall, septum and part of the inferior wall. Mid and right: Short axis apical end diastolic (mid) and end systolic (right) tagging images with application of the semi-automatically traced lines from HARP software. Circumferential mid-wall end systolic strain was calculated from deformation of the orange mid-wall line.

Tagging recordings were analyzed with Harmonic Phase Imaging (HARP version 1.0, Diagnosoft Inc, Palo Alto, California). For each slice, 24 mid-wall points were semi-automatically tracked, and circumferential Lagrangian end systolic strain was calculated from deformation of the line between points. Each of the 24 strain measurements were manually assigned to the 16 segment model[15], and mean strain was obtained for all segments, selected LV regions and the global LV. By convention negative strain indicates myocardial shortening. Typical segmental tagging analysis of an apical slice is illustrated in Figure 1.

Segments were excluded for further analysis if more than half of the segment's strain curves had been excluded due to noise. The LV regions were excluded for further analysis if more than half of the segments in the region had been excluded. Global mean strain values were excluded if more than 50% of the segments had been excluded or if either the basal, mid-ventricular or apical slices suffered from missing segmental data.

LV rotation data were obtained from the mid-wall tracking also made for strain analysis in the basal and apical slices, values given in degrees. LV twist was calculated as basal rotation subtracted from apical rotation. Strain and twist inter- and intra-observer reproducibility was assessed through repeated analyses of 10 randomly chosen tagging examinations.

Late enhancement short axis slices were manually assigned to basal, mid or apical left ventricular slices and divided into sectors to fit the 16 segment model[15]. Myocardial borders and the enhancing areas were manually delineated (PACS, Sectra, Sweden)[12,16-18]. Absolute and relative myocardial infarct volumes were obtained for each segment. Myocardial and infarct masses were converted from volume by multiplying by 1.05 g/ml[19].

LGE and strain data were analyzed globally and in corresponding LV regions, based on baseline LGE findings. All infarct segments in each patient were summarized, denoted infarct zone. All segments without late enhancement were denoted remote segments. Segments with more than 75% infarct were studied selectively, denoted transmural infarct zone, corresponding to the transmurally infarcted segments studied by Herbots et al.[8].

### Statistics

Categorical variables were analyzed with the chi-square test. Continuous data are presented as mean ± standard deviation, and in the gross material all variables for baseline and end-point analyses approximated a normal distribution. For comparisons between groups at baseline two-sample t-tests were performed. Intra-group changes were evaluated by paired sample t-tests. Development between 2-3 weeks and 6 months was assessed by analysis of covariance, with the baseline values used as a covariate. Corresponding analyses were performed on patients with lower than median EF at baseline CMR. Owing to the relatively low number of patients inter-group comparisons were performed with the non-parametric Mann-Whitney test and intra-group change was evaluated with the Wilcoxon signed ranks test. For tagging reproducibility two-way mixed absolute agreement intraclass correlation analyses of global and regional strain and twist were performed. The intraclass correlation coefficients (ICC) are presented with 95% confidence interval. SPSS software version 16.0 was used. Tests were two-sided, and p-values < 0.05 were considered statistically significant.

## Results

### Patient characteristics and LV measurements

Among all patients, the mean age was 58.6 ± 9.1 years, mean time from the onset of symptoms to PCI was 234 ± 104 minutes, and the mean value for maximum creatine kinase MB was 361 ± 136 μg per liter. The characteristics of the patients at admission did not differ significantly between the two groups (Table 1).

**Table 1:** Patient characteristics at admission

	mBMC (n = 15)	Controls (n = 13)	p-value
Age - years	58.5 ± 8.9	58.7 ± 9.7	0.965
Female sex - no	3	3	0.843
Body mass index	25.7 ± 2.6	27.1 ± 3.2	0.240
Current smokers - no	5	3	0.836
Hypertension - no	2	3	0.502
Diabetes mellitus - no	1	1	0.916
Previous angina - no	2	4	0.262
Blood pressure - mm Hg			
systolic	133 ± 21	125 ± 18	0.320
diastolic	79 ± 12	78 ± 13	0.807
Heart rate - beats/min	71 ± 12	80 ± 14	0.090
Time from symptom onset to PCI - min	239 ± 100	228 ± 112	0.780
Maximum creatine kinase MB - μg/l	382 ± 130	334 ± 143	0.376

The results of volumetric, LGE, strain and twist analyses for all patients are summarized in Table 2, and selected results for the group of patients with baseline EF lower than median (51.9%) are summarized in Table 3. At baseline, there were no significant differences between the groups for EF, ESV, EDV, LV mass, infarct size, LV strain or LV twist, and there were no significant differences in myocardial mass, infarct mass, infarct percent or strain in the LV regions examined.

**Table 2:** Global and regional results; all patients (n = 28)

		Baseline			6 months		
		mBMC	controls	p-value 1	mBMC	controls	p-value 2
**Left ventricle** N = 28	EDV - ml	175.1 ± 48.7	176.8 ± 52.8	0.932	174.9 ± 60.0	174.3 ± 62.3	0.828
	ESV - ml	91.2 ± 35.2	84.9 ± 41.0	0.668	87.6 ± 44.9	78.0 ± 42.3	0.674
	EF - %	49.3 ± 9.5	54.1 ± 11.3	0.233	52.4 ± 11.9	57.9 ± 10.1	0.610
	LV mass - g	168.6 ± 28.3	171.5 ± 42.1	0.831	162.4 ± 30.8	160.4 ± 41.1*	0.379
	infarct mass - g	38.6 ± 23.3	45.7 ± 29.5	0.484	37.6 ± 20.5	35.7 ± 20.4*	0.034
	infarct percent - %	22.1 ± 12.4	26.4 ± 14.8	0.410	22.2 ± 10.8	22.0 ± 11.0*	0.026
	global strain - %	-11.1 ± 2.4	-11.9 ± 3.4	0.512	-11.1 ± 2.6	-13.1 ± 2.4*	0.027
	twist - °	11.3 ± 3.6	12.8 ± 5.4	0.365	12.6 ± 4.3	11.8 ± 3.7	0.411

**Infarct zone** N = 27	mass - g	103.0 ± 39.6	103.9 ± 37.6	0.955	100.3 ± 39.9	96.2 ± 34.5*	0.217
	infarct mass - g	38.6 ± 23.3	45.7 ± 29.5	0.484	37.6 ± 20.5	35.7 ± 20.4*	0.034
	infarct percent - %	34.4 ± 13.6	41.5 ± 17.7	0.242	34.3 ± 12.6	35.0 ± 11.6*	0.084
	strain - %	-9.7 ± 3.4	-9.3 ± 4.1	0.770	-10.2 ± 3.3	-11.8 ± 3.0*	0.009

**Transmural infarct zone** N = 13	mass - g	32.3 ± 22.6	35.0 ± 26.2	0.855	33.7 ± 21.4	33.6 ± 24.0	0.519
	infarct mass - g	29.0 ± 23.0	32.5 ± 25.2	0.804	26.6 ± 17.7	26.1 ± 19.5	0.468
	infarct percent - %	86.8 ± 7.4	90.3 ± 6.1	0.377	77.6 ± 4.3*	76.6 ± 11.4*	0.401
	strain - %	-2.0 ± 1.5	-6.6 ± 5.6	0.107	-2.4 ± 1.4	-8.6 ± 4.9	0.108

**Remote segments** N = 27	mass - g	64.4 ± 22.1	66.5 ± 24.3	0.816	61.1 ± 22.8	63.2 ± 24.0	0.965
	strain - %	-14.4 ± 2.4	-14.9 ± 2.3	0.560	-14.3 ± 3.3	-15.6 ± 2.3	0.195

p-value 1 refers to between group differences at baseline (two sample t-test). p-value 2 refers to between group differences in changes from baseline (analysis of covariance). *p < 0.05 for intra-group changes from baseline, evaluated by paired sample t-test.

**Table 3:** Selected results; patients with baseline EF < median (51.9%)

		Baseline			6 months		
		mBMC	controls	p-value 1	mBMC	controls	p-value 2
**Left ventricle** N = 14	EDV - ml	183.2 ± 46.1	202.9 ± 49.3	0.641	194.6 ± 64.4	191.6 ± 55.6	0.162
	ESV - ml	105.1 ± 33.1	114.8 ± 38.1	0.640	107.7 ± 43.4	95.2 ± 43.6*	0.028
	EF - %	43.3 ± 6.4	43.7 ± 7.2	0.947	45.8 ± 7.1	51.9 ± 8.8*	0.386
	infarct percent - %	28.8 ± 10.7	33.5 ± 12.1	0.505	27.2 ± 8.8	28.7 ± 12.1*	0.071
	global strain - %	-10.0 ± 2.4	-11.3 ± 2.9	0.536	-11.4 ± 2.8	-13.0 ± 2.8	0.189

**Infarct zone** N = 14	infarct percent - %	41.0 ± 12.4	46.6 ± 11.9	0.463	37.9 ± 10.1*	38.8 ± 11.4*	0.053
	strain - %	-8.7 ± 2.9	-8.9 ± 2.7	0.738	-9.9 ± 3.1	-11.7 ± 2.4*	0.205

**Transmural infarct zone** N = 9	infarct percent - %	86.8 ± 7.4	90.5 ± 7.7	0.462	77.6 ± 4.3*	79.2 ± 12.7	0.327
	strain - %	-2.0 ± 1.5	-4.6 ± 4.9	0.221	-2.4 ± 1.4	-8.6 ± 5.9	0.027

p-value 1 refers to between group differences at baseline (Mann-Whitney). p-value 2 refers to between group differences in changes from baseline (Mann-Whitney). *p < 0.05 for intra-group differences from baseline, evaluated by Wilcoxon signed rank test.

EDV and ESV did not change over time, but there was a trend towards increased EF from the baseline examination to 6 months, irrespective of treatment allocation. In the subgroup of patients with low baseline EF, EF increased significantly (43.7 ± 7.2 to 51.9 ± 8.8%, p = 0.042) and ESV decreased significantly (114.8 ± 38.1 to 95.2 ± 43.6 ml, p = 0.043) in the control group but did not change in the mBMC group.

### Late gadolinium enhancement

**Figure 2:**
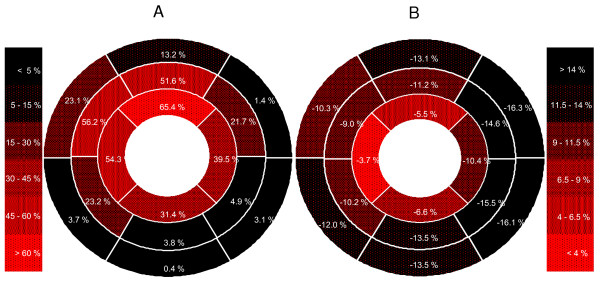
**Bull's eye plots over baseline LGE and strain in the 16 LV segments model**. 2 a: Average infarct involvement (percent) in each segment (n = 448). 2 b: Average strain (percent) in each segment (n = 402). Negative strain values indicate myocardial shortening, and a strain value with amplitude higher than -14% is considered normal.

Average segmental LGE at baseline is illustrated in Figure 2a. All 448 segments in all 28 patients were included for LGE analysis at both time points. The individual LV infarct percent at baseline ranged from 2.2% to 50.1%. Ten patients had small (< 18%), 9 had intermediate (18-30%) and 9 had large infarcts (> 30%)[20]. A total of 271 segments were judged as partially or completely infarcted at baseline, respectively 266 at 6 months. The infarcts affected the basal segments of LV in 15 patients only; 14 of these had large and medium-sized infarcts. In all but 1 patient the infarct involved at least 1 segment outside the presumed LAD supplying territory[15]. In the mid-ventricular part of LV, there was LGE in the inferior septum in 26 patients and in the anterior lateral wall in 23 patients. In the apical part the inferior and lateral segments were infarcted in 27 and 24 patients, respectively. LV infarct mass and infarct percent decreased significantly from baseline to 6 months in the control group only, and there was significant difference between the mBMC and control groups. Regionally, infarct percent decreased significantly in the infarct zone of the control group, and in the transmural infarct zone of both groups. Infarct mass of the infarct zone decreased significantly more in the control group than in the mBMC group. In the group of patients with low baseline EF, infarct percent in the infarct zone decreased more in the controls than in the mBMC patients.

### LV function

Average segmental strain at baseline is illustrated in Figure 2b. Forty-six segments were excluded for strain analysis at baseline, and 35 segments were excluded at 6 months. LV strain was analyzed in 26 patients. Regionally, infarct zone strain and strain of remote segments were analyzed in 27 patients. Thirteen patients had 1 or more segments with more than 75% infarct, and all were included for transmural infarct zone strain analyses. The ICC of agreement for inter-observer judgments was 0.97 (0.87-0.99) for global strain, 0.96 (0.83-0.99) for infarct zone strain and 0.95 (0.65-0.99) for transmural infarct zone strain. Corresponding ICC of agreement for intra-observer observations was 0.99 (0.95-1.00), 0.98 (0.81-0.99) and 0.96 (0.82-0.99) respectively. For individual segments agreement was lower with an ICC of 0.81 (0.75-0.86) for intra-observer and 0.87 (0.83-0.91) for inter-observer judgments.

LV function as measured by global strain improved significantly from baseline to 6 months in the control group (from -11.9 ± 3.4 to -13.1 ± 2.4%, p = 0.014), but remained unchanged in the mBMC group. In the control group, regional myocardial function improved significantly in the infarct zone (from -9.3 ± 4.1 to -11.8 ± 3.0%, p = 0.001), but not in the transmural infarct zone or the remote segments. Regional myocardial function remained unchanged in the mBMC patients. In the group of patients with low baseline EF, strain improvement was significantly more pronounced for the controls in the transmural infarct zone only. LV twist was analyzed in all 28 patients. Twist was low at baseline for both groups, and did not change significantly over time in either of the groups. The ICC of agreement for inter-observer judgments was 0.92 (0.74-0.98) and for intra-observer observations 0.96 (0.85-0.99).

The changes of LV strain in the control group correlated significantly with LV infarction mass at baseline and change of LV infarction mass (Table 4).

**Table 4:** Correlation between strain development and infarct mass in controls

	Beta (standardized)	R	p-value
LV infarct mass at baseline	-0.663	0.663	0.019
Change of infarct mass	0.665	0.665	0.018
Dependent variable: change of LV strain			

Linear regression analysis for correlation between change of LV strain (dependent) and baseline LV infarct mass and change of LV infarct mass in controls (n = 12).

## Discussion

The ASTAMI study, which included 100 patients, was originally designed with a power of 80% to reveal a potential difference of 5% points of LV EF development between the mBMC and the control groups as measured by single-photon-emission computed tomography. No difference was found for global functional development or for clinical parameters[2,21]. For LV EF inter-observer variability was assessed with ICC of 0.85 (0.67-0.93) for FLASH cines and ICC of 0.98 (0.96-0.99) for trueFisp cines[2] (Supplementary Appendix). In this substudy, only 28 patients were included, resulting in reduced power. Unlike previous reports, myocardial wall function developed beneficially for the control group as compared with the mBMC group. Strain improvement was 2.5 ± 2.0% points in the infarct zone and 1.6 ± 1.8% points globally. Infarct mass as shown by LGE developed similarly to strain, with a more pronounced infarct mass reduction for the controls than for the mBMC group. Both groups had a parallel, but statistically non-significant trend towards LV EF improvement. The results from this study illuminate the potentials for detailed regional myocardial examination to detect subtle differences of myocardial function not always detected by other examination techniques routinely used.

Care must be taken drawing conclusions from the results regarding development of myocardial function and LGE in this study. Recently, Beitnes et al. published results from the long-term follow-up of the ASTAMI trial including global and regional LAD strain analyses performed with longitudinal two-dimensional speckle-tracking echocardiography (2D STE)[10]. They reported a significant improvement of regional and global peak systolic strain from baseline to 6 months, which was maintained at three years post-infarction. However, there were no significant differences between the groups regarding change in myocardial systolic or diastolic function. The present substudy introduces some methodological refinement to this study. Circumferential strain was calculated from short axis CMR tagging versus longitudinal strain calculation from long axis 2D STE. For the regional CMR tagging analysis the infarct zone was directly defined by findings from baseline LGE, achieved during the same image session. Inter- and intra-observer strain reproducibility was excellent for global and regional strain analyses, even though reproducibility decreased when strain was studied on the individual segmental level. For the echo examination there was no opportunity to directly delineate the myocardial infarct area, and the region studied was the predefined LAD territory. Additionally, strain calculations were made at different time points. Baseline of the echo study was 4.5 ± 1.1 days after the myocardial infarction, before the bone marrow aspiration and intracoronary mBMC injection of the mBMC group, whereas the baseline CMR examination was performed at 18.8 ± 3.8 days. This fact is also relevant for the comparison of the global LV volumetric analyses. Even though the groups did not develop significantly differently, the CMR examinations in the main ASTAMI study revealed a larger increase of LV EF (1.2% vs 4.3%, p = 0.054), and a larger decrease of infarct size (-2.3 vs -5.9 ml, p = 0.11) in the control group than the mBMC group[2]. There were no between-group differences in baseline infarct mass in the larger, main ASTAMI study. Although not significant, the baseline infarct mass in the substudy was slightly, but not significantly higher in the control group than in the mBMC group. Strain values in the ischemic heart are closely related to LGE[22-24], and strain improves over time after acute myocardial infarction[25]. In the present study, there was a significant correlation between improvement of strain and reduction of infarct size in the control group (Table 4). Part of the differences between the study groups may have been caused by the small study group bias.

In the main ASTAMI study, 72% of the patients who received intracoronary cell injections reported mild chest pain during the procedure and 77% had transient ischemic ST deviation during balloon inflation. No patient had reinfarction related to the procedure[2]. Beitnes et al. recently reported no adverse effects observed after three years in the same population[21]. Thus, there is no indication that the intracoronary balloon inflation procedure has influenced the clinical results of the mBMC group. The present study was not designed to reveal any subtle immediate beneficial or unfavorable changes in the myocardium after intracoronary injection of mBMC. One might speculate, however, that recurrent balloon inflations might negatively influence myocardial function in the treatment perfusion area, as indicated in our results.

Regional wall function has been investigated by different means in other stem cell trials. In the Repair-AMI trial regional function improved more in infarcted areas in the stem cell group judged by the centerline chord method at 4 months[6]. In a recent CMR substudy by Dill et al., infarct area wall thickening was assessed by cine CMR examination, and the results indicated more pronounced increase of wall thickening in the stem cell group after 12 months in the group of patients with initial EF lower than median EF (48.9%)[9]. In the BOOST trial, wall thickening and wall motion in infarcted areas were studied by short axis cine CMR. There was no significant treatment effect from stem cell injection on global LVEF or on the regional function parameters studied at 18 months or 5 years follow up[7,26]. In the trial from Leuven wall thickening was assessed by short axis cine CMR in transmurally and non-transmurally infarcted segments. The change in systolic function did not differ at 4 months between the stem cell group and the placebo group[5]. However, there was a beneficial stem cell treatment effect on strain as measured by tissue Doppler imaging for segments with initial LGE involvement of more than 75%[8]. The results from our substudy do not support any potential beneficial effect from intracoronary injection of mBMC after reperfused myocardial infarction. This also applies to the patients with larger transmural myocardial infarcts and those with low EF.

End systolic LV twist is the difference in the systolic clockwise rotational movement of the basal region from the counter-clockwise rotational movement of the apical region as seen from the apex. The rotation is part of the complex wringing movement of the left ventricle during systole[27,28]. Baseline end systolic LV twist values in our study were 11.3 ± 3.6°and 12.8 ± 5.4°in the mBMC group and controls, respectively. Despite lack of healthy controls for comparison, we regard these values to be clearly lower than the normal values as measured by speckle-tracking echocardiography[29,30]. Reproducibility was excellent, with ICC of 0.92 and 0.96 for inter- and intra-observer variability, respectively. Several authors have found reduced twist in acute or chronic myocardial ischemia compared with twist in healthy individuals, and twist reduction is correlated to the extent of LV EF reduction[29,31,32]. Twist and the more simplified apical torsion have been suggested as reliable and sensitive tools to detect LV dysfunction. In a patient group of acute LAD myocardial infarction, Han et al. found a positive correlation between LV EF and twist, and in addition twist significantly improved one month after revascularization, parallel to a mean EF increase from 38.8% to 49.7%[33]. In the present study mean baseline EF was higher. Further studies, also differentiating smaller from larger infarctions, are needed to evaluate potentials and clinical significance for twist development after acute myocardial infarction.

According to the standardized scheme for myocardial territory assignment the LAD artery supplies the anterior wall and the anterior part of the septum, as well as the apical septum and the apical cap[15]. Ortiz-Pérez et al. have suggested a modification to this scheme, adding the mid anterolateral segment and the apical lateral and inferior segments to the LAD territory[34]. Individual variation will add complexity to these schemes in any patient studies. In the present study, only patients with their first ST-elevation anterior wall infarction were included. Small, medium-sized and large infarcts were quite evenly represented. The relative lack of basal slice LGE in the small infarcts probably was a consequence of the site for LAD occlusion. In the mid-ventricular slices LGE of the anterior lateral wall and the inferior part of the septum was of similar magnitude, and quite significant. Visually, the LGE areas of these segments were fringes of the LGE in the neighboring LAD territory segments. In the apical slices all segments were largely affected, supporting the modification suggested by Ortiz-Pérez et al.[34]. As illustrated in Figure 2 average reduction of segmental strain followed a pattern similar to the average LGE involvement, although strain values were relatively lower in the septum.

## Limitations

The time point for the baseline CMR at least two weeks after the acute event was chosen to reduce overestimation of infarct size owing to tissue edema, and T2-weighted sequences for evaluation of edema were therefore not part of our protocol. Obviously, more information on LV volumes and function could have been available with an additional CMR examination before or immediately after intracoronary cell injection. Thus, we may have missed early and short term changes after the injection, and thereby possible differences between the groups, either related to stem cell effect, or even to the intracoronary balloon inflation procedure itself. This fact also is relevant for the direct comparison between echo and CMR results. However, the time point for baseline CMR examination was suitable for the investigation of myocardial remodeling after the acute phase. Serial CMR studies underscore the advantages of examining LGE after the first 7 days of resorption of necrotic myocardium and hemorrhage, reduction of myocardial edema and possibly rescue of myocardium at risk. A better correlation was found between later functional parameters and LGE after one week compared with LGE immediately after the infarction and reperfusion[35-37].

Short duration of follow-up and a relatively small number of patients, even reduced by exclusions from tagging noise; make the present study insufficient in the assessment of post mBMC treatment effects. LV EF was examined with the area-length method, possibly adding variability compared with multiple short-axis cines. Differences in development may have been masked. The selection of patients did not completely concur with the main ASTAMI study, with a non-significant, numerically slightly higher initial infarct mass in the control group than the mBMC group.

## Conclusions

The results from the present study do not support the hypothesis that intracoronary mBMC injection after reperfused anterior wall infarction reduces infarct size or improves myocardial function. Findings from regional LGE and strain analyses quite similarly demonstrate subtle differences between the mBMC and control groups with a slightly more favorable development in the controls. The potential role for LV twist as assessed by CMR tagging has to be evaluated in further studies.

## Competing interests

The authors declare that they have no competing interests.

## Authors' contributions

EH carried out CMR examinations, image analysis and drafted the manuscript. KL and SS coordinated the study. HA, KF and SA participated in the study design. TE participated in the study design and strain analysis. HJS participated in the study design and CMR analysis. All authors have made revisions to the manuscript and have read and approved the final version.
